# Predictors, Prevalence, and Clinical Outcomes of Out-of-Hospital Cardiac Arrests in Croatia: A Nationwide Study

**DOI:** 10.3390/healthcare11202729

**Published:** 2023-10-13

**Authors:** Damir Vazanic, Biljana Kurtovic, Sasa Balija, Milan Milosevic, Ognjen Brborovic

**Affiliations:** 1 Croatian Institute of Emergency Medicine, 10000 Zagreb, Croatia; sasa.balija@hzhm.hr; 2 Department of Nursing, Catholic University of Croatia, 10000 Zagreb, Croatia; 3 University of Applied Health Sciences, 10000 Zagreb, Croatia; biljana.kurtovic@zvu.hr; 4 Faculty of Health Studies, University of Rijeka, 51000 Rijeka, Croatia; 5 School of Medicine, University of Zagreb, 10000 Zagreb, Croatia; milan.milosevic@snz.hr (M.M.); obrborov@snz.hr (O.B.)

**Keywords:** out-of-hospital cardiac arrest, emergency medical service, resuscitation

## Abstract

Background: Out-of-hospital cardiac arrest (OHCA) remains a pivotal health challenge globally. In Croatia, there has been a knowledge gap regarding the prevalence, predictors, and outcomes of OHCA patients. This study aims to determine the prevalence, prediction, and outcomes of OHCA patients in Croatia. Methods: An extensive one-year analysis was performed on all OHCA treated by the Emergency Medical Service in Croatia, based on the Utstein recommendations. Data were extracted from Croatian Institute of Emergency Medicine databases, focusing on adult individuals who experienced sudden cardiac arrest in out-of-hospital settings in Croatia. Results: From 7773 OHCA cases, 9.5% achieved spontaneous circulation pre-hospital. Optimal outcomes corresponded to EMS intervention within ≤13 min post-arrest onset AUC = 0.577 (95% CI: 0.56–0.59; *p* < 0.001) and female gender OR = 1.81 (95% CI: 1.49–2.19; *p* < 0.001). Northern Croatia witnessed lower success rates relative to the capital city Zagreb OR = 0.68 (95% CI: 0.50–0.93; *p* = 0.015). Conclusions: Early intervention by EMS, specifically within a 13-min period following the onset of a cardiac arrest, significantly enhances the probability of achieving successful OHCA outcomes. Gender differences and specific initial heart rhythms further influenced the likelihood of successful outcomes. Regional disparities, with reduced success rates in northern Croatia compared to the City of Zagreb, were evident.

## 1. Introduction

Out-of-hospital cardiac arrest (OHCA) poses a significant medical challenge with often devastating patient outcomes. Amongst individuals who suffer from OHCA, only 33% are admitted to the hospital, and 8% get discharged [1]. Recognizing the regional variations in OHCA prevalence is crucial as it is influenced by factors including demographic characteristics, availability of emergency medical care, population density, urbanization, and the presence of public defibrillators [2,3,4].

In-depth research has shown that many elements impact OHCA incidence. These elements range from patient-specific factors such as age [5,6], socioeconomic background and overall health condition [7,8], patient needs [9], and established priorities [10] to system parameters like primary healthcare organization [11]. The role of Emergency Medical Services (EMS) is paramount in managing OHCA. Recent advancements in analyzing EMS intervention databases allow researchers to pinpoint system factors that elevate the quality of predictions [12]. These improvements, in line with the Utstein guidelines [13], primarily focus on ensuring swift EMS response, quality of cardiopulmonary resuscitation (CPR), and eventually, enhancing OHCA patient survival rates [14].

When evaluating outcomes post-OHCA, survival rate remains a significant metric. Resuscitation methods have evolved, and timely application of CPR and defibrillation has been linked with better patient outcomes post-OHCA [15]. In Croatia, the EMS system is primarily governed at the county level, where teams respond based on dispatcher instructions. These medical dispatchers hold the responsibility for telephone-guided resuscitation, a method proven to enhance chest compression quality and resuscitation outcomes [16]. EMS in Croatia is organized through 21 county emergency medical institutes. Each county EMS has an associated medical dispatch unit (MDU) where all calls from their respective areas are received and triaged using the Croatian Index for MDU, which dispatches EMS teams to interventions. The emergency medicine network defines the number, distribution, and composition of these teams for each county. The total number of teams per one shift is 183 in Croatia. These teams consist of physicians, nurses, and drivers.

Traditional evaluations of outpatient services have focused on “timeliness” [17,18], but comprehensive quality indicators should be designed for each interval of care [19].

Swor et al.’s findings emphasize the importance of recognizing and addressing the initial rhythms of sudden cardiac arrest, such as ventricular fibrillation and ventricular tachycardia [20]. Sasson et al. further demonstrated that interventions, like layperson resuscitation, provided prior to the return of spontaneous circulation (ROSC) have a higher predictive value concerning OHCA outcomes [21]. The importance of rapid interventions can also be seen in studies showing the benefits of defibrillation within 5 min of an OHCA event [22]. Such quick interventions, along with rapid EMS activation, CPR performance by bystanders, and ROSC in the field, are all consistently linked to better survival rates post-OHCA [23]. However, while these factors are essential, some researchers argue that they do not fully capture the variability in survival outcomes [21].

This is the first OHCA report from Croatia. The aim of this study was to determine the prevalence, prediction, and outcomes of OHCA patients in Croatia.

## 2. Materials and Methods

In Croatia, all occurrences of out-of-hospital cardiac arrest (OHCA) attended to by the EMS within the timeframe spanning from 1 October 2017 to 1 October 2018 were meticulously documented. Adherence to the established Utstein recommendations was maintained during the surveillance of these cardiac arrest episodes. Data acquisition was executed utilizing records from the Croatian Institute of Emergency Medicine database, in conjunction with the standardized Utstein cardiac arrest data collection form. The study’s inclusion parameters encompassed adult individuals who underwent sudden OHCA within Croatian out-of-hospital venues. Exclusionary criteria delineated the omission of individuals aged below 18 years, as well as patients with cardiac arrest precipitated by etiological factors such as trauma, drug overdose, electric shock, lightning impact, drowning, or asphyxiation.

The data are presented in tables and graphs. The distribution of continuous numerical values was analyzed by the Kolmogorov–Smirnov test, and appropriate nonparametric tests were applied according to the obtained data. Categorical and nominal values are shown through the appropriate frequencies and proportions. Continuous values are presented through median and interquartile ranges, and differences between independent groups are analyzed by the Mann–Whitney U test. ROC analysis analyzed individual time intervals to determine the optimal values in the prediction of successful ROSC to the hospital, and the highest values of sensitivity and specificity with the highest values of the Youden index were used as criteria. A binary logistic regression model was made to predict a group of patients who had successful ROSC by the time they arrived at the hospital. *p*-values less than 0.05 were considered significant. Licensed IBM SPSS Statistics software version 25.0 (https://www.ibm.com/analytics/spss-statistics-software (accessed on 26 April 2023)) was used in the analysis.

The study was approved by the Ethics Committee of the Croatian Institute for Emergency Medicine, No. 510-14/16-01/01.

## 3. Results

Descriptive statistics of socio–demographic and clinical characteristics related to arrest in all subjects (N = 7773) is shown in Table 1. Men predominate in almost two-thirds of all respondents: 4825 (62.1%). The most common location of the arrest was the apartment, 5561 (71.5%) cases, while 3847 (49.5%) had witnesses. The cause of arrest in most respondents, 5244 (67.5%), was heart attack.

Yearly incidence is reported as cases per 100,000 persons. Incidence is calculated by dividing the total number of EMS attempted (3460) and each ROSC before reaching the hospital (740) by the total population in Croatia (3,871,833 from 2021 census) and multiplying by 100,000. EMS attempted incidence is 89.36 per 100,000 persons. ROSC before reaching the hospital incidence is 19.11 per 100,000 persons.

Prevalence of specific clinical outcomes among EMS actions during the study is shown in Table 2. Arrest was recognized in only 1726 (22.2%) respondents, while 726 (9.3%) respondents received telephone instructions for resuscitation. Lay resuscitation was attempted in 1640 (22%) subjects, and defibrillation in 1130 (14.5%) subjects. In summary, resuscitation by EMS was attempted in 3460 (44.5%) subjects, and this number was used in subsequent analyses to confirm or refute the hypothesis. The return of spontaneous circulation (ROSC) until arrival at the hospital (measure of the outcome of the immediate EMS resuscitation procedure) was recorded in 740 subjects (9.5% of the total number, or 21.4% of the number of subjects on whom resuscitation was attempted). Most patients, 5120 (65.9%), were involved during the day shift (from 8:00 a.m. to 8:00 p.m.). 

Outcome of resuscitation procedure depending on the specific time intervals relevant to the patients who were in resuscitation is shown in Table 3. Significantly less values in group with successful ROSC before reaching the hospital were found in time from departure to stopping of the vehicle (*p* = 0.004), time from stopping the vehicle to reaching the patient (*p* < 0.001), time from the onset of cardiac arrest to the arrival of the team to the patient (*p* < 0.001), and time from onset of cardiac arrest to first defibrillation (*p* < 0.001).

ROC analysis of successful ROSC prediction in relation to individual time intervals is shown in Table 4. The highest Youden index was used for defining the most optimal values of individual time intervals in the prediction of ROSC success until arrival at the hospital. The value of ≤13 min from cardiac arrest to team arrival to patient had the largest area under the ROC curve (AUC = 0.577) with a sensitivity of 49% and a specificity of 64.01% in the prediction of successful ROSC to hospital arrival.

Table 5 shows a multivariate regression model for predicting a group of patients who had successful ROSC by the time they arrived at the hospital. The regression model is statistically significant (*p* < 0.001) and explains 16.1% of the variance of the dependent variable (ROSC success). From the predictor variables put into the model, the probability of successful ROSC to hospital significantly increases with the time ≤ 13 min from the onset of cardiac arrest to the team’s arrival to patient with an odds ratio (OR) of 1.36 (95% confidence interval (CI) 1.14–1.62; *p* = 0.001), female gender with OR = 1.81 (95% CI: 1.49–2.19; *p* < 0.001) and initial heart rate (relative to asystole as reference value) PEA with OR = 2.41 (95% CI: 1.88–3.10; *p* < 0.001), ventricular fibrillation with OR = 5.81 (95% CI: 4.67–7.23; *p* < 0.001), and ventricular tachycardia with OR = 5.74 (95% CI: 3.59–9.17; *p* < 0.001). The probability of successful resuscitation is significantly reduced by resuscitation in the region of northern Croatia compared to the City of Zagreb with OR = 0.68 (95% CI: 0.50–0.93; *p* = 0.015). Croatia is divided into 4 regions comprising 21 counties, as shown in the table. 

## 4. Discussion

Our study provided essential insights into the demographics and clinical characteristics of patients who experienced out-of-hospital cardiac arrest in Croatia. Key findings encompass the male predominance, the prevalence of cardiac arrests occurring within residences, and the significant influence of time intervals on successful resuscitation.

In aligning our findings with the extant literature, our study corroborates previously reported gender differences in OHCA. The study by Christiansen et al. [24] demonstrated a notably higher incidence of cardiac arrest among males, consistent with our observations. Furthermore, the nationwide data from Norway further supports our results, presenting a higher incidence rate of heart failure in males across all age groups [25]. Interestingly, a meta-analysis by Feng et al. [26] highlighted that, despite being older, less likely to experience arrest in public places, exhibiting less initial shockable rhythm, and being less likely to be witnessed by bystanders or provided with CPR, women still exhibited a significant survival advantage post-OHCA. These gender disparities emphasize the necessity for tailored therapeutic strategies and public health interventions, considering the unique clinical presentations and outcomes associated with each gender. 

The location of the OHCA event has been the subject of extensive research, given its potential implications for response strategies and outcomes. In our study, a notable majority of OHCAs occurred within residential settings, with 5561 out of 7773 cases (71.5%) taking place in apartments. This trend seems consistent with the findings from the North American population where, out of 12,930 evaluated OHCAs, 9564 occurred at home [27]. Interestingly, Borgstedt et al. (2023) found that the incidence of return of spontaneous circulation (ROSC) did not vary significantly between public and non-public locations (*p* = 0.4). However, patients experiencing OHCA in public spaces were more frequently admitted to the hospital with spontaneous circulation (*p* = 0.011) [28]. The probability of successful ROSC to hospital significantly increasing is also shown in our study by initial heart rate (relative to asystole as reference value) PEA with OR = 2.41 (95% CI: 1.88–3.10; *p* < 0.001), ventricular fibrillation with OR = 5.81 (95% CI: 4.67–7.23; *p* < 0.001), and ventricular tachycardia with OR = 5.74 (95% CI: 3.59–9.17; *p* < 0.001). Weisfeldt (2011) reported that the occurrence of initial ventricular fibrillation or pulseless ventricular tachycardia, key determinants of OHCA outcomes, were considerably higher in public settings than at home. This disparity suggests that the inherent advantages of certain resuscitation strategies, such as the immediate availability of an AED, might be influenced by the location of the cardiac arrest [27]. The observed variations based on arrest location highlight the importance of context-specific preparedness and interventions in the management of OHCA.

Building upon the gender disparities and the environment of out-of-hospital cardiac arrest onset, it is crucial to emphasize that regardless of the aforementioned factors, the universal tenet remains consistent in highlighting the pivotal role of a prompt response in the management and outcome of these patients. This importance is highlighted across multiple studies conducted worldwide, underscoring its global significance. One study reported that between 2014 and 2017, out of 12,073 cases, 723 EMS responses related to OHCA were analyzed. Shockable initial heart rhythm, defibrillation, and resuscitative efforts initiated by an emergency physician were found to significantly enhance the chances of a patient’s successful admission to a hospital with spontaneous circulation [28]. This underlines the necessity for early recognition and intervention in OHCA cases. Similarly, a Swedish study by Holmen et al. revealed that the ambulance response time for OHCA in the country has doubled over the past three decades. However, survival chances following an OHCA have seen a significant upswing during the same period. They further emphasized that survival to 30 days post a witnessed OHCA dropped as ambulance response times increased [29]. Our research also underscores the same point. The time of ≤13 min from cardiac arrest to team arrival was crucial in predicting successful ROSC upon hospital arrival. Specifically, the probability of successful ROSC at the hospital increased significantly when the response time was ≤13 min, with an odds ratio of 1.36. In a study from Serbia, the authors noted that initiating CPR within the first 4 min post OHCA significantly elevated survival rates. They stressed the importance of minimizing emergency response times to further enhance these survival outcomes [30]. Furthermore, technological advancements are now being proposed to further optimize response times. For instance, Bogle et al. suggest employing drones equipped with automatic external defibrillators to ensure rapid defibrillation, which could drastically boost OHCA survival rates [31]. Similarly, a study on helicopter emergency medical services in the UK highlighted the potential of such services in the management of OHCA, even though they were never the first to arrive on the scene [32]. Time intervals, especially the time from the onset of cardiac arrest to the EMS’s arrival, are crucial in predicting successful resuscitation.

Our study underscored the vital role of timeframes in relation to the geographical distribution and accessibility of EMS. Specifically, our findings elucidated that the likelihood of successful resuscitation diminishes considerably in the region of northern Croatia as opposed to the City of Zagreb, with an odds ratio of 0.68 (95% CI: 0.50–0.93; *p* = 0.015). Supporting our observations, a systematic review by Alanazy et al. meticulously examined the disparities between urban and rural EMS settings [33]. Adhering to PRISMA guidelines, this review utilized a rigorous search strategy across multiple databases. The eventual conclusions drawn from 31 relevant studies underscored the superior performance measures of urban EMS, reflected by reduced prehospital times, quicker response durations, and higher survival rates in cases of out-of-hospital cardiac arrests or trauma when juxtaposed against their rural counterparts. A striking revelation was the dearth of studies from low and lower-middle-income nations, emphasizing the need for more granular research in these regions to bridge the evident gap. Mell et al. indicated that while the average EMS response time after a 911 call is about 7 min in urban settings, it can exceed 14 min in rural areas [34]. Given these findings, it is imperative to address temporal and regional disparities to enhance patient outcomes and streamline EMS operations.

The interpretation of our findings has certain limitations. Primarily, our study relies on register data, which inherently poses the risk of misclassification, particularly concerning the performance of bystander CPR, encompassing both rescue breaths and chest compressions. Additionally, our dataset lacks medical history data and comprehensive information elucidating the reasons bystanders refrained from administering CPR.

## 5. Conclusions

This study revealed significant determinants influencing the success of resuscitation outcomes. A timely response, specifically a ≤13-min interval from cardiac arrest onset to EMS team arrival, emerged as a pivotal factor in predicting successful ROSC before hospital admission. Gender differences and specific initial heart rhythms further influenced the likelihood of successful outcomes. Notably, regional disparities, with reduced success rates in northern Croatia compared to the City of Zagreb, were evident. Thus, it is recommended for EMS systems to prioritize rapid response measures, particularly in identified high-risk areas, to optimize resuscitation outcomes in OHCA incidents.

## Figures and Tables

**Table 1:** Descriptive statistics of socio–demographic and clinical characteristics related to arrest in all subjects (N = 7773).

		N	%
Gender	Male	4825	62.1
	Female	2948	37.9
Arrest location	Ambulance	34	0.4
	Motorway	14	0.2
	Road	205	2.6
	Care home	533	6.9
	Public space	65	0.8
	Educational institution	2	0
	Other	376	4.8
	Open public space	730	9.4
	Workplace	28	0.4
	Sports and recreational facility	9	0.1
	Flat	5561	71.5
	Enclosed public space	216	2.8
Witnessed arrest	No witnesses	2286	29.4
	Unknown	901	11.6
	Eyewitness	3847	49.5
	Team EMS	739	9.5
Pathogenesis	Asphyxia	165	2.1
	Medical	568	7.3
	Other	1259	16.2
	Overdose	27	0.3
	Heart attack	5244	67.5
	Electric shock	6	0.1
	Traumatic	416	5.4
	Lightning strike	1	0
	Drowning	87	1.1
Age	Mean (SD)	70.5	15.4

**Table 2:** Prevalence of specific clinical outcomes among EMS actions during the study.

		N	%
Arrest recognized	Yes	1726	22.2
Dispatcher provided CPR instructions	Yes	726	9.3
Bystander response	Yes	1640	22
First monitored rhythm	Asystole	5840	75.1
	PEA	803	10.3
	VF	1036	13.3
	VT	94	1.2
Defibrillation by EMS team	Yes	1130	14.5
Resuscitation attempted	No attempt was made	1259	16.2
	No attempt was made—signs of death present	2943	37.9
	Not attempted—circulation signs present	111	1.4
	Attempted	3460	44.5
Airway control	Yes	3401	43.8
Vascular access	Yes	3256	41.9
Survived event	Yes	740	9.5
Spontaneous breathing after ROSC	Yes	433	5.6
Conscious after ROSC	Yes	116	1.5
Shift	Day shift	5120	65.9
	Night shift	2653	34.1

**Table 3:** Outcome of resuscitation procedure depending on the specific time intervals relevant to the patients who were in resuscitation (N = 3460): Mann–Whitney U test.

	ROSC before Reaching the Hospital	N	Min	Max	Median	Percentile 25	Percentile 75	*p*
Time from receiving the call to sending the team (min)	Unsuccessful	2720	0.0	79.0	2.0	1.0	2.0	0.172
	Successful	740	0.0	56.0	1.0	1.0	2.0	
Time from departure to stopping of the vehicle (min)	Unsuccessful	2720	1.0	65.0	7.0	4.0	12.0	0.004
	Successful	740	1.0	60.0	7.0	4.0	11.0	
Time from stopping the vehicle to reaching the patient (min)	Unsuccessful	2720	0.0	44.0	0.0	0.0	1.0	<0.001
	Successful	740	0.0	18.0	0.0	0.0	1.0	
Time from the onset of cardiac arrest to the arrival of the team to the patient (min)	Unsuccessful	2720	1.0	100.0	16.0	11.0	24.0	<0.001
	Successful	740	1.0	81.0	13.5	9.0	20.0	
Time from onset of cardiac arrest to first defibrillation (min)	Unsuccessful	2720	2.0	75.0	18.0	13.0	25.0	<0.001
	Successful	740	1.0	78.0	16.0	12.0	22.0	
Time from departure from the place of intervention to arrival at the hospital (min)	Unsuccessful	2720	2.0	86.0	13.0	7.0	23.0	0.057
	Successful	740	2.0	106.0	12.0	6.0	20.5	
Time from receiving the call to the arrival of the patient (min)	Unsuccessful	2720	0.0	97.0	12.0	8.0	17.0	0.051
	Successful	740	0.7	95.0	11.0	7.0	16.0	

**Table 4:** ROC analysis of successful ROSC prediction in relation to individual time intervals.

	AUC	95% CI	Criterion	Sensitivity	Specificity	*p*
Time from receiving the call to sending the team (min)	0.517	0.501 to 0.534	≤1	21.35	82.39	0.147
Time from departure to stopping of the vehicle (min)	0.536	0.519 to 0.552	≤7	54.32	52.13	0.003
Time from stopping the vehicle to reaching the patient (min)	0.539	0.522 to 0.556	≤1	45.14	62.10	<0.001
Time from the onset of cardiac arrest to the arrival of the team to the patient (min)	0.577	0.560 to 0.593	≤13	49.05	64.01	<0.001
Time from onset of cardiac arrest to first defibrillation (min)	0.565	0.535 to 0.594	≤17	56.02	55.32	<0.001
Time from departure from the place of intervention to arrival at the hospital (min)	0.532	0.504 to 0.560	≤9	38.92	69.15	0.056
Time from receiving the call to the arrival of the patient (min)	0.524	0.507 to 0.540	≤17	79.19	25.96	0.049

**Table 5:** Prediction of successful ROSC before reaching the hospital according to relevant time intervals: binary logistic regression.

	OR	95% CI		*p*
		Lower	Upper	
≤13 min time from the onset of cardiac arrest to the arrival of the team to the patient	1.36	1.14	1.62	0.001
Age (years)	0.99	0.99	1.01	0.051
Region: City of Zagreb (ref.value)				0.060
Adriatic Croatia	0.86	0.63	1.16	0.324
Northern Croatia	0.68	0.50	0.93	0.015
Panonic Croatia	0.78	0.56	1.09	0.143
Female gender	1.81	1.49	2.19	<0.001
Phone resuscitation instructions given	1.13	0.84	1.52	0.426
Arrest recognized	0.91	0.72	1.15	0.440
Heart rhythm at arrest location: asystole (ref.value)				<0.001
PEA	2.41	1.88	3.10	<0.001
VF	5.81	4.67	7.23	<0.001
VT	5.74	3.59	9.17	<0.001
Lay revival	0.83	0.67	1.03	0.093
Night shift	0.88	0.73	1.06	0.185

## Data Availability

The data presented in this study are available on request from the corresponding author.
